# First CpG island microarray for genome-wide analyses of DNA methylation in Chinese hamster ovary cells: new insights into the epigenetic answer to butyrate treatment

**DOI:** 10.1186/1753-6561-7-S6-O5

**Published:** 2013-12-04

**Authors:** Anna Wippermann, Sandra Klausing, Oliver Rupp, Thomas Noll, Raimund Hoffrogge

**Affiliations:** 1 Cell Culture Technology, Bielefeld University, Bielefeld, Germany; 2 Center for Biotechnology, Bielefeld University, Bielefeld, Germany

## Background

Optimizing productivity and growth of recombinant Chinese hamster ovary (CHO) cells requires insight and intervention in regulatory processes. This is to some extent accomplished by several 'omics' approaches. However, many questions remain unanswered and bioprocess development is therefore still partially empirical. In this regard, the analysis of DNA methylation as one of the earliest cellular regulatory levels is increasingly gaining importance. This epigenetic process is known to influence transcriptional events when it occurs at specific genomic regions with high CpG frequencies, called CpG islands (CGIs). Being methylated, CGIs attract proteins with methyl-DNA binding domains (MBD proteins) that in turn can interact with chromatin modifying complexes, thereby leading to a transcriptionally inactive state of the associated gene [1]. In CHO cells, DNA methylation has yet only been investigated in gene-specific approaches, e.g. regarding the CMV promoter [2]. To analyze differential DNA methylation in CHO cultures on a genomic scale, we developed a microarray covering 19,598 CGIs in the CHO genome. We applied it to elucidate the effect of butyrate on CHO DP-12 cultures, as this short chain fatty acid (SCFA) is known to elicit epigenetic responses by inhibiting histone-deacetylases [3].

## Materials and methods

Based on the genomic and transcriptomic information available for CHO cells [4,5], 21,993 promoter-associated and intragenic CGIs were identified in the CHO genome using an algorithm according to Takai and Jones [6]. We developed a customized 60K microarray (printed by Agilent Technologies) covering 19,598 (89%) of the identified CGIs with an average probe spacing of 500 bp. Genomic DNA of each four replicate experimental and reference CHO DP-12 (clone #1934, ATCC CRL-12445) batch cultures was phenol-chloroform extracted and sheared by sonication. Methylated fragments were enriched using the methyl-CpG binding domain of MBD2 protein fused to the Fc tail of IgG1 (MBD2-Fc protein) coupled to magnetic beads (New England Biolabs). Experimental samples prior to treatment with 3 mM butyrate (0 h) as well as 24 hours and 48 hours after butyrate addition were directly compared to the references by two-colour co-hybridizations. Data analysis was carried out upon LOWESS normalization by Student's t-tests with p-values ≤ 0.05 using the open source platform EMMA2 [7]. Confirmatory COBRA (combined bisulfite restriction analysis) was performed by amplifying a 541 bp fragment of the myc proto-oncogene protein-like gene (Gene ID: 100758352) following bisulfite treatment of genomic DNA using the primers myc_for 5'-atttggaaggatagtaagtatattggaag-3' and myc_rev 5'- aaataaaactctaactcaccatatctcct-3' and the nested primers myc_for_nested 5'- atagtaagtatattggaaggggagtg-3' and myc_rev_nested 5'- taaaactctaactcaccatatctcctc-3' (oligonucleotides obtained from Metabion). Purified PCR products were digested with BstUI (Fermentas) and separated in agarose gels.

## Results

**Figure 1:**
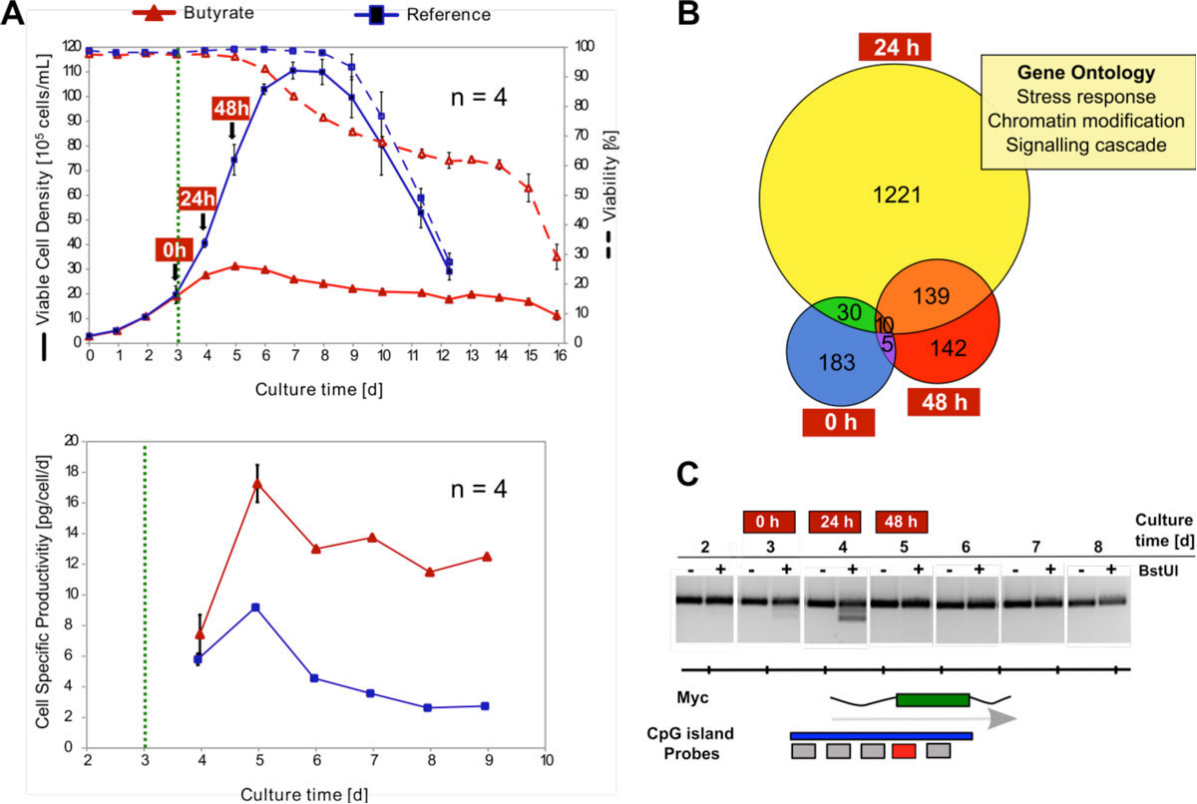
**(A) Viable cell densities, viabilities and cell specific productivities for batch CHO DP-12 reference (blue) and butyrate treated (red) cultivations**. The green dashed line marks the point of butyrate addition. Error bars represent standard deviations. **(B) **Venn diagram showing the numbers of genes associated with differentially methylated CpG islands before (0 h), 24 hours and 48 hours upon butyrate addition. Gene Ontology classification was performed using DAVID [9] with an EASE score ≤ 0.01 **(C) **COBRA analysis of a part of the CGI (blue) of the myc proto-oncogene protein-like gene (green) differential methylation was detected for (red). Cleavage products indicate methylation of BstUI sites in the original DNA.

Butyrate treated CHO DP-12 cultures stopped proliferating and decreasing viabilities could be detected 24 hours upon addition of the SCFA (Figure 1A). Simultaneously, cell specific productivities increased by nearly 100 % (17 pg/cell/day 48 hours after butyrate addition compared to 9 pg/cell/day in the reference cultures). Surprisingly, 228 differentially methylated genes could be detected in a comparison between the experimental cultures and the references even before addition of butyrate (Figure 1B), indicating substantial heterogeneity among identically handled parallel cultivations. 24 hours after butyrate addition we found a strongly increased number of 1221, solely at this point in time, differentially methylated genes. Gene ontology classification showed that, amongst others, the terms 'stress response', 'chromatin modification' or 'signalling cascade' were significantly overrepresented. Pathways such as the Ca^2+^, MAPK and Wnt signalling systems were comprised within the latter group and showed a large coverage by differentially methylated components. 48 hours upon butyrate addition the number of differential methylations decreased by about 90 %. COBRA analysis of the Wnt responsive myc proto-oncogene protein-like gene showed clearly detectable cleavage products (indicating methylation of the BstUI sites in the original DNA) 24 hours upon butyrate addition, that completely vanished another 24 hours later (Figure 1C), confirming the results of the microarray analysis.

## Conclusions

Our first genome-wide screening for differential DNA methylation in CHO cells shows that the epigenetic response upon butyrate treatment seems to be highly dynamic and reversible. This was confirmed by applying the bisulfite-based single-gene method COBRA to analyze a region of the myc proto-oncogene protein-like gene. Furthermore, detection of differential methylation before butyrate addition indicates that heterogeneity in DNA methylation occurs even if cells originated from the same preculture and were treated identically. This occurrence of differentially methylated genes in parallel cultivations strongly fosters the hypothesis that the culture history influences final process outcomes [8]. It underlines the importance of DNA methylation analyses in CHO cells, especially considering the fact that DNA methylation patterns can remain stably anchored over several generations.
